# Serum Peroxiredoxins Reflect Oxidative Stress and Predict Renal Outcomes in Patients with Glomerulonephritis

**DOI:** 10.3390/ijms26167708

**Published:** 2025-08-09

**Authors:** Natalia Wiewiórska-Krata, Barbara Moszczuk, Julia Tańska, Emilia Knioła, Ewelina Grywalska, Leszek Pączek, Bartosz Foroncewicz, Krzysztof Mucha

**Affiliations:** 1 Laboratory of Cellular and Genetic Therapies, Center for Preclinical Research, Medical University of Warsaw, 02-006 Warsaw, Poland; 2 ProMix Center (ProteogenOmix in Medicine), Department of Clinical Immunology, Medical University of Warsaw, 02-006 Warsaw, Poland; 3 Department of Clinical Immunology, Medical University of Warsaw, 02-006 Warsaw, Poland; 4 Department of Transplantology, Immunology, Nephrology and Internal Diseases, Medical University of Warsaw, 02-006 Warsaw, Poland; 5 Department of Experimental Immunology, Medical University of Lublin, 20-093 Lublin, Poland; 6 Institute of Biochemistry and Biophysics, Polish Academy of Sciences, 02-106 Warsaw, Poland

**Keywords:** biomarkers, chronic kidney disease, oxidative stress, peroxiredoxins, prognosis

## Abstract

Oxidative stress (OS), defined as an imbalance between pro-oxidant and antioxidant mechanisms, contributes to DNA and protein oxidation as well as cellular injury, and plays a pivotal role in the pathogenesis of chronic kidney disease (CKD). Peroxiredoxins (PRDXs) are key antioxidant enzymes that regulate intracellular peroxide levels and maintain redox homeostasis. Beyond its renal implications, OS is closely intertwined with hypertension and atherosclerosis, both common comorbidities that accelerate CKD progression. As previously reported, serum concentrations of PRDXs 1-5 may help to differentiate between IgA nephropathy (IgAN), membranous nephropathy (MN), and lupus nephritis (LN). This study aimed to assess the utility of baseline serum PRDX levels in predicting longitudinal changes in kidney function and proteinuria in patients with IgAN, MN, and LN. We analyzed data from 80 patients (IgAN, n = 36; MN, n = 23; LN, n = 21) drawn from an initial cohort of 108 in whom baseline serum concentrations of PRDX 1–5 were measured. Patients were stratified into low, medium, and high PRDX level groups at baseline, and associations between these strata and longitudinal changes in eGFR and proteinuria were assessed over a follow-up period of up to five years. Across all groups, the follow-up eGFR was significantly associated with low baseline serum PRDX 1, 2, 3, and 5 (*p* = 0.043; *p* = 0.001; *p* = 0.036; *p* = 0.007, respectively). Significant associations were also observed between 24 h follow-up proteinuria and low baseline serum PRDX 2, 3, and 5 (*p* = 0.025; *p* = 0.025; *p* = 0.005, respectively), medium PRDX 4 (*p* = 0.010), and high PRDX 2 (*p* = 0.019). No significant associations were found within the study groups; however, these associations were more pronounced in IgAN and MN patients. These findings suggest a potential role for PRDXs in predicting and monitoring CKD progression, especially eGFR decline.

## 1. Introduction

Chronic kidney disease (CKD) is a major public health problem worldwide, since it affects 8–16% of the population [1,2]. Its main consequences include increased risk of cardiovascular disease (CVD), a significant increase in mortality, and a decrease in health-related quality of life [3]. CKD may progress to end-stage kidney disease which is associated with further increased risk of CVD and mortality. In 2018, the leading causes of end-stage kidney disease were diabetes (39%), hypertension (26%), and glomerulonephritis (GN) (15%). The symptoms of GN are mostly nonspecific, and patients may present with proteinuria, erythrocyturia or hematuria, edema, or hypertension. Therefore, diagnosis is challenging and frequently delayed. Moreover, it currently requires histopathologic evaluation of a renal biopsy, which is an invasive procedure and carries obvious risks [4]. Thus, safer, simpler, and quicker diagnostic and monitoring methods are sought.

Several markers are helpful in the diagnosis of GN. For example, galactose-deficient IgA1 and IgG autoantibodies in IgA nephropathy (IgAN) [5], anti-phospholipid 2 receptor antibodies in membranous nephropathy (MN), and cell-free DNA [6] or anti-double-stranded DNA antibodies in lupus nephritis (LN) [7]. Multiple other urine and serum biomarkers [4,8,9,10] or gene polymorphisms [11,12] have been proposed for different forms of GN in the last decade. However, their diagnostic utility remains uncertain.

Recently, oxidative stress (OS), defined as a disequilibrium between the synthesis and neutralization of reactive oxygen species (ROS), was identified as one of the key factors in the pathogenesis of CKD. Exogenous induction of ROS may come from ultraviolet light, other types of radiation, and chemical exposure, or from viral and bacterial infections. Endogenous sources include excessive ROS formation in the mitochondria or the endoplasmic reticulum [13]. If OS is not properly regulated it may initiate harmful effects and contribute to CKD progression [14]. Since kidneys are highly metabolically active and rich in mitochondrial oxidation reactions, they may become vulnerable to ROS damage [15]. Indeed, OS is a nontraditional risk factor for all-cause mortality in CKD and end-stage kidney disease patients [16]. OS involves numerous pathways; therefore, multiple OS indicators are available. They include the peroxiredoxin (PRDX) family of enzymes, which reduce OS by eliminating more than 90% of cellular peroxides [17,18]. Nevertheless, the role of PRDXs in the pathophysiology and progression of GN is largely unknown. We have previously reported that PRDX concentrations vary depending on the GN type and, thus, may be involved in the pathophysiology of IgAN, MN, and LN, indicating that PRDXs may be used in noninvasive GN diagnostics [19]. The aim of the current study was to evaluate the utility of PRDXs measured at baseline in predicting CKD progression in patients with IgAN, MN, or LN.

## 2. Results

Complete follow-up data were available for 80 out of 108 patients. The remaining 28 patients were lost to follow-up. The reasons for loss to follow-up included absence from outpatient appointments (lack of any medical results) during the last 2 years, which may have been largely caused by the COVID-19 pandemic or a change in medical center or place of residence. It was reported that among the lost to follow-up group, 4 patients had renal replacement therapy, resulting in renal transplantation in 2 patients. Additionally, 1 patient died due to ischemic stroke.

Among the complete follow-up participants, the mean ages of female (n = 46) and male (n = 34) GN participants were 50.5 and 51.7 years, respectively. Disease distribution was as follows: IgAN (n = 36), LN (n = 21), and MN (n = 23). The patient clinical characteristics are summarized in Table 1.

Multiple regression analysis revealed the significant associations of follow-up eGFR (R^2^ = 0.286, F = 4.876, *p* = 0.0003) in terms of gender, age, prevalence of anemia, hypertension, and dyslipidemia. The detailed regression coefficients (Table 2) represent the relative strength of predictors, which was the highest in HT (−0.248) and dyslipidemia (−0.224), resulting in *p*-values of 0.022 and 0.034, respectively.

**Figure 1:**
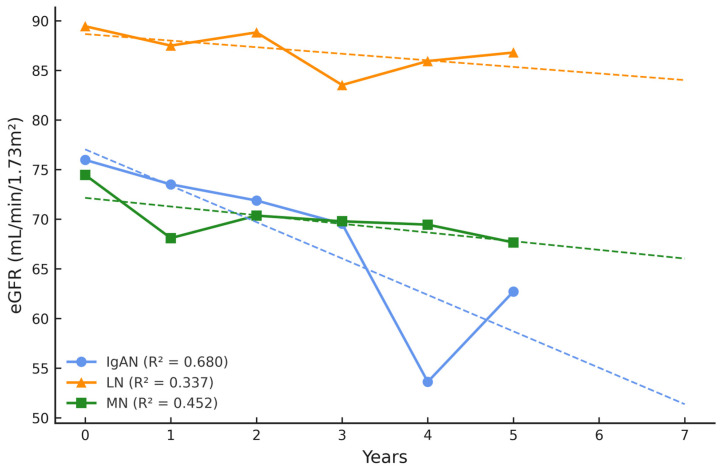
eGFR during 5-year follow-up and 2-year prognosis.

The majority of patients (71%) had an eGFR ≥ 60 mL/min per 1.73 m^2^ at baseline. Interestingly, during the 5-year follow-up we observed that eGFR deteriorated in patients with IgAN or MN, while those with LN were relatively stable. The highest eGFR fluctuations and deterioration were present in the IgAN group (Figure 1), and doubling of serum creatinine (DSC) was reported in three patients (two IgAN and one MN).

**Figure 2:**
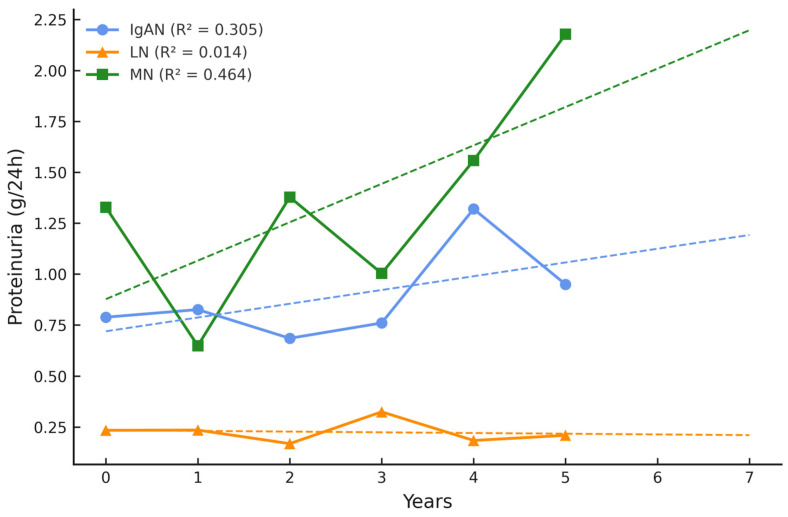
Proteinuria during 5-year follow-up and 2-year prognosis.

At baseline, most of the patients exhibited 24 h proteinuria levels below 1 g/day. Notably, over the 5-year follow-up period, proteinuria tended to increase in patients with MN and, to a lesser extent, in those with IgAN, whereas individuals with LN generally maintained stable urinary protein excretion. The highest 24 h proteinuria fluctuations were present in the MN group (Figure 2).

Moreover, the 2-year prognosis (dashed line) indicates that the eGFR will continue to decline in the IgAN group and proteinuria will progress in the MN group. Notably, no significant disease flares were observed in these patient groups.

When the analysis included all GN patients, their eGFR value after 5 years was significantly associated with a baseline serum concentration of PRDX 1, 2, 3, or 5 below the 20th percentile (*p* = 0.043, *p* = 0.001, *p* = 0.036, and *p* = 0.007, respectively, Table 3). PRDX concentrations higher than the 20th percentile had no significant relation to eGFR after 5 years.

Significant associations were also observed between the 24 h proteinuria after 5 years in all GN patients and low serum PRDX 2, 3, or 5 (*p* = 0.025, *p* = 0.025, and *p* = 0.005, respectively), medium PRDX 4 (*p* = 0.010), and high PRDX 2 (*p* = 0.019) concentrations (Table 4).

These results suggest a possible association between serum PRDX concentrations at baseline and the deterioration of renal function over a period of 5 years, especially in IgAN and MN patients.

Additionally, no significant differences in baseline parameters such as age, eGFR, 24 h proteinuria, and PRDX 1, 2, 3, 4, or 5 concentrations (*p* = 0.289; *p* = 0.088; *p* = 0.626; *p* = 0.829; *p* = 0.053; *p* = 0.698; *p* = 0.559; *p* = 0.246, respectively) were found between the completed and lost to follow-up groups, suggesting a low risk of attrition bias.

We also investigated whether disease duration could affect eGFR progression. We found that patients with a longer disease history had stable eGFR during follow-up. However, we can only refer this observation to the 5-year follow-up and not to the entire treatment period.

## 3. Discussion

The main finding of the current study is the significant association between baseline PRDX levels and renal function values after 5 years in stable patients with IgAN, MN, or LN. In particular, low serum concentrations of PRDX 1, 2, and 5 were related to eGFR decline at year 5 of follow-up. Accordingly, an increase in 24 h proteinuria at year 5 was associated with low PRDX 2, 3, and 5 at baseline. These findings suggest a potential role for these PRDXs in the biology of GN and, thus, in predicting and monitoring CKD progression, particularly in IgAN and MN patients.

There is growing evidence that CKD is an inflammatory disorder [20,21,22]. Chronic processes driven by accumulated uremic toxins, OS, and dysregulation of the microbiota activate inflammatory pathways, subsequently increasing proinflammatory cytokines and inflammatory markers [23]. Moreover, OS and inflammation are implicated not only in the initiation, but also in the ongoing progression of CKD [24]. Therefore, OS markers are expected to be potential biomarkers for assessing the risk of CKD progression. Importantly, our study relies on the measurement of the biomarkers at a single time point. Although intra-individual PRDX variations are largely unknown, significant redox status variability has been reported even in healthy subjects [25]. Furthermore, GN patients who are persistently exposed to low-level inflammation may encounter both intra- and inter-individual variations. For this reason some authors propose the use of single measurements of inflammatory biomarkers to predict only short-term outcomes [26]. Nevertheless, numerous studies aiming to predict mortality risk in CKD patients have been designed on the basis of single time point assessments. For example, Wei and colleagues explored the relationship between the baseline SIRI index (neutrophil count × monocyte count/lymphocyte count) and 15-year all-cause and cardiovascular mortality in CKD patients [27]. Other studies by Yoshitomi et al. and Kim et al. revealed that a high baseline neutrophil:lymphocyte ratio is predictive of adverse outcomes in stable CKD patients after 8 years [28,29]. Also, our previous studies associated single measurements of the inflammatory markers interleukin-6 and osteoprotegerin with 5-year risk of all-cause mortality in CKD patients [30,31].

All these reports, along with our current findings, have several important implications. First, they suggest a need for a paradigm shift in CKD monitoring, focusing on not only renal function, but also on systemic inflammation, including OS. Second, they pinpoint the utility of a single measurement of a given biomarker at baseline in predicting long-term CKD outcomes. Furthermore, these studies revealed that CKD patients exhibiting higher inflammatory biomarker levels show a significantly increased risk of CKD progression and related mortality. Consistent with those findings, the results of our current study implicate the predictive role of low levels of protective OS markers in CKD progression. PRDXs are thiol-dependent peroxidases that decompose hydrogen peroxide, lipid hydroperoxides, and peroxynitrite, thus protecting against oxidative and inflammatory stress. Therefore, their low levels may be considered harmful [19,32]. While PRDXs (especially PRDX 1–3) play key roles in detoxifying peroxides in the cytosol and mitochondria, insufficient PRDX expression has been associated with mitochondrial dysfunction, ATP depletion, and enhanced ROS production via NADPH oxidase activation, thereby amplifying oxidative damage within renal tissue [33]. We hypothesize that inadequate concentrations of PRDXs may predict long-term exposure to chronic OS and inflammation, followed by CKD progression. To verify this, repeated long-term evaluation of both PRDXs and ROS simultaneously with markers of inflammation and renal function would be mandatory.

The experimental models described by Choi et al. [34] have elucidated the role of PRDX 5, demonstrating that its deficiency exacerbates angiotensin II-induced hypertension and renal fibrosis through activation of the WNK4-SPAK/OSR1-NCC signaling cascade. Moreover, considering the pivotal role of redox-sensitive transcription factors such as Nrf2, reduced PRDX activity may impair antioxidant gene expression while simultaneously promoting the activation of profibrotic signaling pathways, including NF-κB and TGF-β, ultimately contributing to nephron loss and extracellular matrix deposition [35].

One could also speculate on the impact of the timing of PRDX concentration measurements. For example, PRDX 4 has been independently associated with an increased risk of new onset of CKD in a population-based cohort study [36], while PRDX 1 has been shown to promote inflammation via the NF-κB signaling pathway [37]. In addition, PRDX 2 supports CD8^+^ T-cell responses, which are necessary for regulating immunity and mediating survival during chronic infection [38]. Thus, it is very likely that PRDXs may be elevated at disease onset due to acute oxidative stress and immune activation, while later in the disease course their levels may stabilize at lower concentrations, only to rise again during disease flares, infections, or surgical interventions. In the current study, the patients with GN were diagnosed several years before enrollment, and PRDX measurements were performed in stable chronic conditions. This context may partially explain the lower PRDX levels observed in some individuals and warrants consideration when interpreting their prognostic significance.

Interestingly, the concentrations of various PRDXs differ among patients with different forms of GN. We reported previously that the PRDX pattern may be used to distinguish between IgAN, MN, and LN [19]. Here, our results also support the role of PRDXs in CKD progression. It is known that the rate of progression depends not only on the GN type, but also on the CKD stage. LN patients enrolled in the study appeared to have better renal function at baseline. This may explain the fact that no significant declines in eGFR were detected during the 5-year follow-up in this subgroup of participants. Accordingly, their baseline degree of proteinuria was lower than that of the IgAN and MN patients. These findings explain the differences in the predictive value of PRDXs among the groups. The follow-up period included the COVID-19 pandemic, which could partially explain the differences in CKD progression between the GN types, particularly as IgAN is known to be exacerbated and progress during any type of infection. Of note, we found significant differences between the GN subgroups in terms of gender, age, time from diagnosis, follow-up eGFR, steroid use, and the prevalence of anemia, atherosclerosis, and dyslipidemia. These clinical disparities are important, as they may directly influence systemic inflammatory processes and OS pathways. Given that standard pharmacologic management with renin-angiotensin-aldosterone inhibitors, while essential for reducing intraglomerular pressure and proteinuria, does not fully address persistent OS and endothelial dysfunction, there is a compelling rationale to explore complementary strategies. Although our study design did not include an evaluation of patients’ dietary habits, this aspect warrants attention in the context of oxidative balance. For example, dietary antioxidants, such as polyphenols from berries, may play a meaningful role in modulating OS and cardiovascular risk in patients with glomerular diseases. Kasprzak-Drozd et al. demonstrated that black chokeberry (*Aronia melanocarpa*), rich in anthocyanins and proanthocyanidins, exerts potent antioxidant and anti-inflammatory effects, improves lipid metabolism, and enhances endothelial function—mechanisms directly relevant to the cardiorenal continuum [39]. These findings highlight the potential value of incorporating plant-derived antioxidant strategies alongside standard pharmacologic interventions to mitigate the residual oxidative stress observed in this patient population. However, as underscored in our previous report [40], the unsupervised use of dietary supplements remains a concern, with over 70% of patients with CKD of various etiologies reporting supplement intake without medical consultation. Such practices may inadvertently affect oxidative stress profiles, confound biomarker data, and complicate the interpretation of clinical outcomes.

Collectively, these molecular and clinical insights suggest that low serum PRDX levels are consistent with antioxidant system exhaustion, predisposing individuals to chronic inflammation, endothelial dysfunction, and fibrosis. These findings are consistent with our longitudinal observations in patients with IgAN, MN, and LN.

The study has certain limitations. First, the relatively small number of patients may have biased the results. However, the investigation offers the advantage of comparing patients with different types of GN, including MN and LN, which are registered as rare diseases in the ORPHA database (N° 97560 and 536, respectively) [41,42]. This study was designed as an exploratory investigation to assess the prognostic utility of serum PRDX isoforms in distinct glomerular disease subtypes. Although the total sample size of 80 patients may be considered limited, it reflects the clinical challenge of enrolling well-characterized individuals with biopsy-proven GN and complete 5-year follow-up data. Although we agree that stratification into disease subgroups may reduce statistical power, our findings offer valuable preliminary insights into the relationship between oxidative stress biomarkers and renal outcome trajectories. Second, the MN group was significantly older, and this factor could exacerbate atherosclerosis, dyslipidemia, and oxidative damage in this cohort. Third, the follow-up time can be considered both a limitation and an advantage of our study. On the one hand, a 5-year follow-up is a long enough period to determine clinical outcomes such as CKD progression. On the other hand, markers of OS are at higher risk of fluctuating. These potential fluctuations may be driven by infections or changes in immunosuppressive therapies. The infections were not analyzed in detail due to the study design, which precluded accurate collection of such clinical data. However, patients were followed to record significant GN exacerbations. Since we did not detect any, we consider the patients stable with respect to natural GN progression. The fourth limitation of our study is that we measured the PDRXs’ concentrations, not their activities. Therefore, we may not have gained a complete insight into the true activity of oxidative stress.

## 4. Materials and Methods

This was a prospective 5-year follow-up study of 108 GN patients who were primarily recruited in 2017 [19], according to the following criteria: lack of active infection, malignancy, previous organ transplantation, or current pregnancy. The study was approved by the Ethics Committee of the Medical University of Warsaw (N^o^ KB/9/2010 and KB/199/2016). Informed consent was obtained from all participants involved in the study.

The clinical and biochemical parameters of CKD progression were monitored for 5 years between 2017 and 2022 at 12 (±3)-month intervals and collected based on the medical records from outpatient visits. The patients were considered lost to follow-up if they did not show up for their appointments in the last 2 years of observation. Complete follow-up data were available for 80 out of 108 patients. The following parameters were evaluated at baseline and during follow-up visits.

### 4.1. Baseline

The serum creatinine concentration, complete blood count (CBC), urine analysis, and 24 h proteinuria were assayed by routine laboratory techniques using automatic analyzers (Cobas Integra 400 Plus and Elecsys 2010; Roche Diagnostics, Mannheim, Germany). The estimated glomerular filtration rate (eGFR) was calculated according to the Chronic Kidney Disease–Epidemiology Collaboration equation.

The routine laboratory tests were performed in the diagnostic laboratory of Infant Jesus Hospital, University Medical Centre of the Medical University of Warsaw, during routine patient visits to the Nephrology and Transplantation Outpatient Clinic.

PRDX concentrations were measured with commercially available enzyme-linked immunosorbent assays (EIAab, Wuhan, China), as described previously [19].

### 4.2. Follow-Up

The serum creatinine concentration, CBC, urine analysis, and 24 h proteinuria were assayed at every outpatient visit. Additionally, a 2-year prognosis beyond the 5-year follow-up was estimated using linear regression.

**Figure 3:**
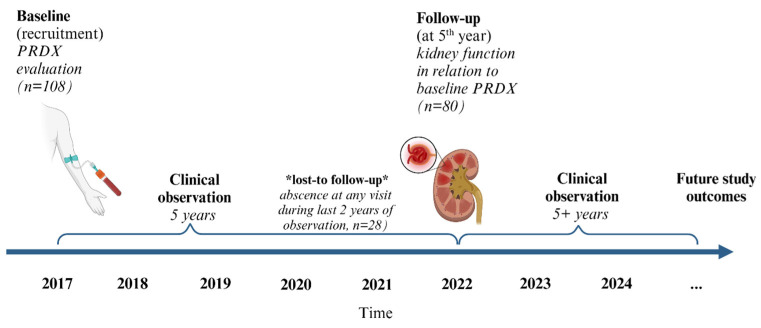
Timeline of study observation.

The timeline of study observations over the 5-year follow-up period is presented in Figure 3.

In order to investigate the association between baseline serum PRDX concentrations and longitudinal changes in renal function parameters (eGFR and proteinuria), patients were stratified into three groups based on the distribution of baseline PRDX levels: low (≤20th percentile), medium (20th–80th percentile), and high (>80th percentile). The thresholds for each PRDX isoform are detailed in Table 5.

**Figure 4:**
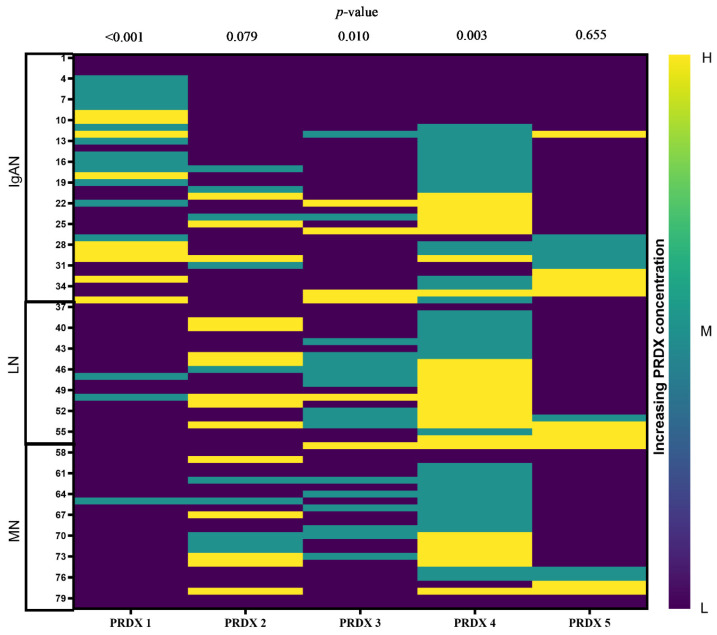
Heatmap of PRDX concentration panels for 80 individuals who completed 5-year follow-up. Abbreviations: left panel, patient IDs; right panel, L—low, M—medium, H—high PRDX concentration; *p*-values were calculated with Kruskal–Wallis test.

The baseline PRDX concentrations of patients who completed the 5-year follow-up are presented in Figure 4.

The follow-up eGFR and proteinuria values were analyzed on the basis of PRDX concentrations in the whole GN group and in each GN subgroup.

### 4.3. Statistical Analysis

All statistical analyses were performed using Statistica version 13.3 (TIBCO Software Inc., Palo Alto, CA, USA). Continuous variables are presented as the mean ± standard deviation or as the median with interquartile range, as appropriate. Normality was assessed using the Shapiro–Wilk test. Non-normally distributed variables were analyzed with the Kruskal–Wallis or Mann–Whitney U tests. Categorical variables were compared using the chi-square test. Linear regression was employed to estimate trends in eGFR and proteinuria within GN subgroups over time. Associations between changes in eGFR (dependent variable) and selected clinical or biochemical predictors (independent variables) were evaluated using multiple regression analysis. A *p*-value < 0.05 was considered statistically significant. Figures were prepared with GraphPad Prism 10.2.3 and BioRender.com.

## 5. Conclusions

In summary, our results suggest a need for a paradigm shift in CKD monitoring, to focus not only on renal function, but also on systemic inflammation and oxidative stress. The results highlight the need for personalized treatment strategies guided by biomarker profiling and emphasize the potential utility of PRDXs as prognostic markers in glomerular diseases. In particular, low serum concentrations of PRDX 1, 2, 3, and 5 may help to predict CKD progression in IgAN and MN patients.

## 6. Patents

This project is a continuation of a study on the role of PRDXs in GNs, the results of which have been granted patent No. EP3358355.

## Figures and Tables

**Table 1:** Clinical characteristics of patients with complete 5-year follow-up data.

GN, n = 80	IgAN, n = 36	LN, n = 21	MN, n = 23	*p*-Value
Gender (F/M)	20/16	16/5	10/13	0.024
Age, years	47.11 (13.0)	48.90 (12.1)	58.96 (13.9)	0.010
Time from diagnosis, years	15.6 (10.3)	9.2 (4.4)	16.1 (7.9)	0.002
Serum creatinine, mg/dL at baseline	1.22 (0.6)	0.88 (0.3)	1.12 (0.5)	0.079
Serum creatinine, mg/dL at follow-up	1.63 (1.2)	0.90 (0.3)	1.35 (1.1)	0.005
eGFR (mL/min/1.73 m^2^) at baseline	75.98 (31.0)	89.46 (28.7)	74.46 (25.7)	0.181
eGFR (mL/min/1.73 m^2^) at follow-up	62.71 (30.6)	86.81 (25.8)	67.65 (27.7)	0.013
Δ eGFR (mL/min/1.73 m^2^)	−13.27 ↓	−2.65 ↓	−6.81 ↓	n.a.
eGFR slope (mean)	−0.21	−0.02	−0.07	n.a.
24 h urine protein (g/24 h) at baseline	0.79 (0.6)	0.23 (0.2)	1.33 (2.2)	0.305
24 h urine protein (g/24 h) at follow-up	0.95 (0.99)	0.21 (0.2)	2.18 (2.8)	0.760
Δ 24 h urine protein (g/24 h)	0.16 ↑	−0.02 ↓	0.85 ↑	n.a.
Medications, n				
ACEI	29	16	15	0.410
ARB	8	3	11	0.029
Immunosuppression	3	15	12	<0.001
Steroids	11	7	17	0.002
Comorbidities, n				
Hypertension	32	20	22	0.435
Diabetes mellitus	2	0	2	0.409
Anemia	1	6	1	0.040
Atherosclerosis	1	3	6	0.029
Dyslipidemia	23	7	21	<0.001

Abbreviations: ACEI, angiotensin-converting-enzyme inhibitors; ARB, angiotensin II receptor blockers; eGFR, estimated glomerular filtration rate (CKD-EPI equation); F, female; M, male; GN, glomerulonephritis; values in brackets, standard deviation; Δ, difference between value at last follow-up and baseline; n.a., not available. Values are presented as means. SI conversion factors: to convert creatinine to µmol/L, multiply by 88.4020; for eGFR to mL/s, multiply by 0.0167.

**Table 2:** Multiple regression analysis of follow-up eGFR and selected variables in all subjects.

**Parameter**	**Partial** **Regression Coefficient (β)**	**Standard Error** **(S.E.)**	***p*** **-Value**
Age	−0.216	0.116	0.067
Gender	−0.198	0.101	0.055
HT	−0.248	0.106	0.022
Anemia	−0.049	0.102	0.633
Atherosclerosis	−0.018	0.109	0.867
Dyslipidemia	−0.224	0.103	0.034

**Table 3:** *p*-values calculated with Kruskal–Wallis test for differences between follow-up eGFR (after 5 years) in all GN patients, categorized by baseline PRDX levels.

PRDX Level	PRDX 1	PRDX 2	PRDX 3	PRDX 4	PRDX 5
Low	0.043	0.001	0.036	0.184	0.007
Medium	0.441	0.870	0. 270	0.050	n.a.
High	n.a.	0.052	0.772	0.313	0.640

Abbreviations: Low, (≤20th percentile); Medium, (20th–80th percentile); High, (>80th percentile); n.a.—not available.

**Table 4:** *p*-values calculated with Kruskal–Wallis test for associations between 24 h proteinuria after 5 years in all GN patients, categorized by baseline PRDXs levels.

PRDX Level	PRDX 1	PRDX 2	PRDX 3	PRDX 4	PRDX 5
Low	0.937	0.025	0.025	0.286	0.005
Medium	n.a.	0.342	0.670	0.010	n.a.
High	n.a.	0.019	0.319	0.227	n.a.

Abbreviations: Low, (≤20th percentile); Medium, (20th–80th percentile); High, (>80th percentile); n.a.—not available.

**Table 5:** PRDX stratification based on serum concentration percentiles.

Level	PRDX 1 (pg/mL)	PRDX 2 (ng/mL)	PRDX 3 (ng/mL)	PRDX 4 (pg/mL)	PRDX 5 (ng/mL)
Low	≤15.6	≤0.156	≤0.312	≤78	≤0.78
Medium	15.7–30.46	0.157–0.94	0.312–0.55	78–376.18	0.78–2.04
High	>30.46	>0.94	>0.55	>376.18	>2.04

Abbreviations: Low, (≤20th percentile); Medium, (20th–80th percentile); High, (>80th percentile).

## Data Availability

The data presented in this study are available on request.
